# Is Higher α-Linolenic Acid Content Preferable? Assessing Digestive Fate of Flaxseed Oil in *In Vitro* Digestion Models of Infants, Adults and the Elderly

**DOI:** 10.3390/foods15162891

**Published:** 2026-08-18

**Authors:** Lei Mu, Baijun Chu, Jiangxu Zhang, Ju Hui, Xiangyu Wang

**Affiliations:** 1 Nutrition & Health Research Institute, COFCO Corporation, Beijing 102209, China; mulei1@cofco.com (L.M.); chubaijun@cofco.com (B.C.); zhangjiangxu@cofco.com (J.Z.); huiju@cofco.com (J.H.); 2 Beijing Key Laboratory of Nutrition & Health and Food Safety, Beijing 102209, China; 3 Beijing Engineering Laboratory of Geriatric Nutrition & Foods, Beijing 102209, China

**Keywords:** α-linolenic acid, *in vitro* digestion, infants, elderly, flaxseed oil

## Abstract

α-Linolenic acid (ALA) is an essential omega-3 polyunsaturated fatty acid, yet how dietary ALA enrichment interacts with population-specific gastrointestinal physiology to dictate bioaccessibility and lipid peroxidation remains insufficiently understood. This study systematically evaluated the *in vitro* digestive behaviors and oxidation risks of flaxseed oils containing 52% and 68% ALA using simulated models representative of distinct population groups (infants, adults, and the elderly). Digestive efficiency peaked in the adult model, achieving ALA bioaccessibility of 49.2% (52% ALA oil) and 46.5% (68% ALA oil), significantly outperforming those observed in infants (23.1% and 19.5%) and the elderly (39.4% and 37.3%). Crucially, malondialdehyde (MDA) accumulation directly correlated with digestive lipolysis efficiency across population groups, reaching its maximum in adults (5.00 nmol/mL for 52% ALA oil; 7.54 nmol/mL for 68% ALA oil), followed by the elderly (3.06 nmol/mL and 4.02 nmol/mL), and remaining lowest in infants (0.92 nmol/mL and 1.49 nmol/mL). Within each population cohort, although the 68% ALA oil yielded a higher absolute ALA release amount, its relative bioaccessibility was slightly lower while generating elevated MDA levels. These findings demonstrate that increasing ALA concentration imposes a critical trade-off between lipolytic efficiency and oxidative safety across different population groups, providing a solid scientific rationale for designing population-tailored flaxseed oil products optimized for both nutrient delivery and safety.

## 1. Introduction

Alpha-linolenic acid (ALA) is an essential omega-3 polyunsaturated fatty acid that the human body is unable to produce on its own, necessitating its intake through food sources [1]. It functions as a key precursor in the metabolic pathway leading to the formation of longer-chain omega-3 derivatives, such as eicosapentaenoic acid (EPA) and docosahexaenoic acid (DHA) [2,3]. Flaxseed oil stands out among plant-derived oils for its exceptionally high ALA content, which generally falls between 50% and 70% [4,5]. Physiologically, regular and moderate intake of flaxseed oil has been linked to several beneficial effects, including the reduction in inflammation, modulation of blood pressure, and enhancement of insulin responsiveness [6,7,8,9,10]. In addition, ALA is critically involved in the development of the nervous system in fetuses and young children, while also supporting cognitive performance throughout adulthood and in aging individuals [11,12,13].

However, elevated concentrations of ALA in flaxseed oil do not automatically translate into enhanced nutritional value. One key factor is that during the digestion of dietary lipids, increased fatty acid unsaturation is strongly associated with a reduced rate of fatty acid liberation [14,15,16]. Owing to its high number of carbon-carbon double bonds, flaxseed oil rich in ALA undergoes slower digestive breakdown when compared to less unsaturated fats, including rapeseed oil, sunflower oil, palm oil, and lard [15,16]. Additionally, the digestive system harbors various pro-oxidant elements—such as reactive oxygen species and transition metal ions—and the low pH conditions in the stomach can accelerate lipid peroxidation [17]. Findings from an *in vitro* simulation by Kenmogne-Domguia and colleagues indicate that lipids with greater unsaturation produce higher levels of oxidative degradation products, including malondialdehyde (MDA), in the digestive milieu [18]. These oxidation-derived compounds not only diminish the bioavailability of polyunsaturated fatty acids but may also elevate the susceptibility to chronic disorders such as cardiovascular disease, diabetes, and specific forms of cancer [19,20].

At present, research on the digestive behavior of flaxseed oil has primarily concentrated on variations in lipid molecular structure—such as triacylglycerols (TAG) versus diacylglycerols (DAG) [21]—comparative fatty acid profiles across different oils—including flaxseed, rapeseed, and palm oils [15,16]—and strategies involving delivery systems like microencapsulation or nanoparticles to enhance the oxidative stability and bioaccessibility of ALA during digestion [22,23,24]. In contrast, investigations specifically addressing the influence of varying ALA content within flaxseed oil remain limited, particularly with regard to systematic evaluations of how such variation affects lipid digestion in vulnerable populations like infants and older adults. Infants possess immature digestive systems characterized by low levels of digestive enzyme activity and secretion, while the elderly often experience age-related declines in gastrointestinal function, including reduced digestive capacity and dysbiosis of the gut microbiota [25,26,27]. As a result, both groups exhibit markedly distinct lipid digestion and absorption efficiencies compared to healthy adults, underscoring the need for targeted studies on ALA-rich oils in these populations.

This study examined two different flaxseed oil types with varying ALA levels, using *in vitro* digestion systems to assess their digestive performance under physiological conditions representative of distinct population groups. The study aims to determine the fatty acid composition and glyceride profile before digestion, monitor the release of non-esterified fatty acids (NEFA) and dynamic changes in glyceride profiles during digestion, and evaluate both the bioaccessibility of ALA following digestion and the alterations in MDA concentration. The results provide a scientific foundation and empirical data to support precision nutrition strategies involving flaxseed oil across diverse populations and further offer insights for the development of functional lipid products tailored to the specific physiological requirements of targeted populations.

## 2. Materials and Methods

### 2.1. Materials

Cold-pressed first-grade flaxseed oils with ALA contents of 52% and 68% were purchased from a local supermarket in Beijing. Rabbit gastric extract (RGE-15, measured lipase activity of (10.2 ± 0.3 U/mg), using tributyrin as substrate) was purchased from Lipolytech Company (Marseille, France). Pancreatic lipase (measured lipase activity (61.2 ± 0.6 U/mg), using tributyrin as substrate) and bile salts were purchased from Sigma-Aldrich (New York, NY, USA). Thirty-seven fatty acid methyl esters (FAMEs) and monoglyceride of linolenic acid were obtained from the AMPEL Laboratory Technologies Inc. (Shanghai, China). The PES needle filter (0.45 μm) and the nylon syringe filter (0.22 μm) were purchased from AMPEL Laboratory Technologies Inc. (Shanghai, China). All inorganic chemical reagents were purchased from Sinopharm Chemical Reagent Co., Ltd. (Beijing, China). Isopropyl alcohol, methanol, acetonitrile, pyridine and ammonium acetate are all LC-MS-grade reagents purchased from Thermo Fisher Scientific (Waltham, MA, USA), while the rest are chromatographic grade reagents purchased from AMPEL Laboratory Technologies Inc. (Shanghai, China).

### 2.2. In Vitro Digestion

Prior to digestion, lipids must be emulsified. The PBS solution (pH = 6.8) is formulated by mixing a 5 mM potassium dihydrogen phosphate solution with a potassium hydrogen phosphate solution. Weigh precisely 8.85 g of the PBS solution and 0.15 g of Tween 20 into a 50 mL beaker. Stir (RCT Basic, IKA, Staufen im Breisgau, Germany) the mixture for 30 min, and then add 1 g of flaxseed oil. Shear the mixture for 2 min using a high-speed shear homogenizer (T25, IKA, Germany) at 10,000 revolutions per minute (rpm) [16,28].

According to the INFOGEST 2019 protocol and previous literature [28,29], the SSF, SGF and SIF were prepared on the same day using the method of Mulet-Cabero et al. with only minor adjustments made. Then, 10 mL of oil emulsion was mixed with an equal proportion of simulated oral fluid (containing 1.5 mM CaCl_2_); the pH was adjusted to 6.8. The mixture was stirred at 37 °C for 2 min to simulate the oral digestion process. Subsequently, in the simulated gastric digestion stage, 10 mL of the oral digestion product was mixed with simulated gastric fluid (containing 0.15 mM CaCl_2_) in an equal proportion. At this time, the simulated digestive fluid contained 60 U/mL of rabbit gastric extract (RGE), and the pH was immediately adjusted to 3.0. It was then incubated at 37 °C for 2 h to simulate the gastric digestion process. Finally, in the simulated intestinal digestion stage, 10 mL of the gastric digestion product was mixed with simulated intestinal fluid (containing 0.6 mM CaCl_2_) in an equal proportion. At this time, the simulated digestive fluid contained 10 mmol/L of bile salts and 2000 U/mL of pancreatic lipase, and the pH was rapidly adjusted to 7.0, followed by incubation at 37 °C for 2 h to simulate the intestinal digestion process. Taking into account the distinct physiological characteristics of infants and the elderly, the parameters of the *in vitro* digestion model were specifically refined to better reflect their unique digestive conditions. The digestive systems of infants are not yet fully developed, resulting in lower digestive enzyme activities and insufficient secretion of bile salts. According to Menard et al., the gastric lipase concentration was reduced to 20 U/mL and the pH was adjusted to 5.0 during the gastric digestion phase to simulate the characteristically milder gastric environment of infants [30]. During the intestinal digestion phase, lipid hydrolysis in the small intestine is not entirely absent but rather proceeds with restricted efficiency. Previous studies have shown that lipase release in infants is only 5–6% of that in healthy adults, and their trypsin and bile salt concentrations are far below adult levels, standing at 90 U/mL and 3.1 mM, respectively [25,26,27]. Therefore, the pancreatic lipase concentration was set to 160 U/mL, the bile salt concentration was set to 3 mM, and the pH was maintained at 6.6 to represent the immature intestinal digestive state. By significantly reducing the concentrations of lipolytic enzymes and bile salts, this model provides a biologically relevant simulation of the gastrointestinal fate of infant lipids.

The parameter settings for the elderly *in vitro* digestion model are primarily grounded in the physiological shifts in exocrine decline and gastrointestinal motility reduction. The content of gastric lipase in the bodies of the elderly is approximately 15% of that in adults, while the concentrations of trypsin, pancreatic lipase and bile salts are all 50% of those of healthy adults [27,31]. With advancing age, the general decline in physical peristalsis and mechanical shearing within the gastrointestinal tract significantly slows down food transit and gastric emptying [27]. For the elderly model, gastric lipase concentration was decreased to 9 U/mL, and the duration of the gastric digestion phase was extended to 2.5 h to account for delayed gastric emptying. In the intestinal phase, pancreatic lipase concentration was increased to 1000 U/mL, bile salt concentration was raised to 5 mM, and the incubation time was prolonged to 3 h. To further capture the more alkaline intestinal conditions in older adults, the calcium chloride concentrations in the gastric and small intestinal fluids were increased to 0.3 mM and 1.2 mM, respectively (approximately twice the levels of the adult model). Through these population-specific parameter adjustments, the *in vitro* digestion static model can more accurately simulate the gastrointestinal digestive environment of different populations, providing a more reliable experimental platform for studying the digestive differences in flaxseed oil across different populations.

The *in vitro* lipid digestion process was simulated using a pH-stat (T5, Mettler Toledo, Greifensee, Switzerland), and the amount of released NEFA was determined based on the consumption of 0.1 M NaOH.

(1) $$NEFA\;(\%)=\frac{V_{NaOH}\times C_{NaOH}\times M_{Lipid}\times 100}{W_{Lipid}\times 2}$$

where *V_NaOH_* and *C_NaOH_* represent the consumed volume and molar content of the NaOH, respectively, *M_Lipid_* represents the average molar mass of oil, and *W_Lipid_* represents the total weight of lipid initially present in the reaction vessel [32].

During the simulated small intestinal digestion process, the released NEFA gradually accumulate over time until the maximum total release amount (*Φ_max_*) is reached. The release kinetic parameters of NEFA are calculated using the following formula.

(2) $$\ln\left[{(\Phi }_{max}-\Phi_{t})/\Phi_{max}\right]=-kt+b$$

Here, *k* represents the first-order kinetic rate constant of NEFA release (s^−1^), t represents the duration of the small intestinal digestion process (s), and *Φ_max_* can be obtained from Equation (1). By plotting the curve of ln[(*Φ_max_* − *Φ_t_*)/*Φ_max_*] over time, a first-order linear equation is obtained, from which the kinetic constant k can be calculated [28].

### 2.3. Fatty Acid Composition Analysis by GC

Fatty acid determination was performed using Agilent 7890B gas chromatography (7890B, Agilent, Santa Clara, CA, USA), equipped with a flame ionization detector (FID) and a Merck SP-2560 fused-silica capillary column (100 m × 0.25 mm inner diameter, 0.2 μm film thickness). The fatty acid composition of the undigested fat samples was quantitatively determined according to the area percentage method specified in GB 5009.168-2016 [33], and the fatty acids were preliminarily identified by comparison with a mixed standard containing 37 fatty acid methyl esters (FAMEs). Briefly, 60.0 mg of oil sample was weighed into a centrifuge tube. The sample was dissolved in 4 mL isooctane and methylated by adding 200 μL of 2 mol/L KOH in methanol. After vigorous shaking for 30 s, the mixture was allowed to stand until it became clear. Subsequently, approximately 1 g of anhydrous sodium bisulfate was added to neutralize residual base, and the mixture was vortexed (VRX, IKA, Staufen im Breisgau, Germany) vigorously for 1 min. After phase separation and salt precipitation, the upper organic layer was filtered through a 0.22 μm nylon syringe filter (organic-compatible) and collected for GC injection.

### 2.4. Glyceride Analysis by UHPLC–Q/Orbitrap

Triacylglycerol was analyzed by ultra-high performance liquid chromatography (UHPLC) using a CAPCELL PAK ADME column (2.1 mm × 100 mm, 2.0 μm particle size, 40 °C), coupled to a quadrupole/orbitrap high-resolution mass spectrometry (Q-Exactive, Thermo Fisher Scientific, Waltham, MA, USA) [34,35]. Mobile phase A consisted of water/methanol/acetonitrile (60:20:20, *v*/*v*/*v*), and mobile phase B was isopropanol; mobile phase A contained 5 mmol/L ammonium acetate. The flow rate was set at 0.2 mL/min, with a 5 μL injection volume. The elution gradient program was as follows: 0–1 min, 45% B; 1–15 min, increase to 80% B; 15–17.5 min, increase to 95% B; 17.5–17.6 min, return to 45% B; 17.6–25 min, hold at 45% B.

Identification of glycerol esters was performed in positive electrospray ionization (HESI+) mode. Mass spectrometric conditions were as follows: ion source temperature: 350 °C; capillary heating temperature: 320 °C; sheath gas (N_2_) flow rate: 40 L/min; auxiliary gas (N_2_) flow rate: 15 L/min; spray voltage: 3.00 kV. Data were acquired in parallel reaction monitoring (PRM) mode, and the resolution was set at 17,500. Stepped normalized collision energy (NCE) was applied at 10%, 15%, and 25%, with an isolation window of 1.0 m/z. Identification of triacylglycerols (TAGs) was achieved by matching observed precursor exact masses against the target mass spectrometry list (Table S1), maintaining a mass error threshold within 10 ppm. Quantification of TAGs was performed using the peak area normalization method. Extracted ion chromatograms (XICs) of precursor ions were integrated using Xcalibur 2.1 software (Thermo Fisher Scientific), and the relative abundance of each TAG species was expressed as its peak area percentage relative to the total peak area of all identified TAGs.

### 2.5. Acylglycerol Profile Analysis by GC

Lipids in the digestion mixture were extracted using a solvent mixture of n-hexane/methanol/formic acid (1:1:0.1, *v*/*v*/*v*), following protocols adapted from the Folch method, and the Bligh and Dyer method [36,37]. This solvent system was selected to simultaneously recover both nonpolar and polar lipid components. The acylglycerol composition of the extracted lipids was analyzed by gas chromatography (GC-2030, Shimadzu, Kyoto, Japan) equipped with a flame ionization detector (FID) and an Agilent DB-1ht column (15 m × 0.25 mm inner diameter, 0.1 μm film thickness). The operating procedure was adapted according to the actual situation. Briefly, the oil sample (100–200 mg) was accurately weighed into a test tube, to which 4 mL of pyridine solution was added. The mixture was vortexed to ensure complete homogenization, then filtered into a 2 mL vial, and the filtrate was collected for further use.

Then, 50 μL of the filtrate was transferred to a 2 mL screw-cap vial, and 250 μL of silylation reagent (99% BSTFA, 1% TCMS) was added. The vial was tightly capped, shaken vigorously, and incubated (Herathem OGH60, Thermo, Waltham, MA, USA) at 70 °C for 20 min to complete derivatization. The resulting solution was then injected into the GC system for analysis [38]. The data were processed using the area percentage method, and quantitative analyses were conducted on the digested TAG, DAG, MAG and NEFA.

### 2.6. Analysis of ALA Content in Micelles

In contrast to the undigested oil, intestinal digestion hydrolyzes triacylglycerols into NEFA and monoglycerides, which are subsequently solubilized within mixed micelles. The bioaccessibility of lipophilic bioactive components is normally defined as the fraction that is solubilized within the mixed micelle phase after lipid digestion [39]. Following intestinal digestion, the samples were centrifuged at 10,000 rpm for 30 min at 4 °C (Refrigerated centrifuge, Xiangyi, Shanghai, China). The micellar layer was then collected for fatty acid determination. The lipids in the micellar layer were extracted from the digesta using n-hexane/methanol/formic acid (1:1:0.1, *v*/*v*/*v*). The upper hexane phase was dehydrated over anhydrous sodium sulfate and transferred to glass vials with PTFE-lined septa. As the lipid components of the micelles contain NEFA and monoglycerides, acid-catalyzed methylation (5% sulfuric acid in methanol, 70 °C, 60 min) is applied to ensure the complete esterification of the two lipids. Fatty acid methyl esters were extracted with 1 mL of n-hexane, filtered through a 0.22 μm nylon syringe filter, and analyzed by gas chromatography. The contents were quantified using an external calibration method, with monolinolenin serving as the external standard. This method can perform absolute quantification of total ALA in the micellar phase, including NEFA and MAG binding forms, thereby comprehensively reflecting the total amount of bioaccessibility ALA available for intestinal absorption.

The bioaccessibility rate can be calculated using the following formula:

(3) $$Bioaccessibility\left(\%\right)=\frac{C_{1}\times V_{total}}{C_{0\times m}}\times 100\%$$

where *C*_1_ is the mass concentration of ALA in 1 mL micellar phase aliquot (mg/mL); *V_total_* is the total volume of the micellar phase (mL); *C*_0_ is the relative ALA content in the undigested oil sample (mg/g); and *m* is the mass of the undigested oil sample (g).

### 2.7. MDA Content Determination

Add 10 mL of n-hexane to the digestive fluid, vortex for 2 min, then centrifuge (Multifuge X1 Pro, Thermo, Waltham, MA, USA) at 10,000 rpm for 5 min. Transfer the entire lower aqueous phase supernatant to another centrifuge tube, mix well, and take an appropriate amount of the clear liquid to pass through a 0.45 μm PES membrane. According to the instructions of the kit, determine the MDA level in the lower clear liquid.

### 2.8. Data Analysis

All experiments were performed with at least three independent biological replicates, and data were expressed as mean ± standard deviation. Statistical analysis was conducted using R 4.5.0 (R Foundation for Statistical Computing, Vienna, Austria) operated via RStudio 2025.09.1 (Posit Software, Boston, MA, USA). One-way analysis of variance (ANOVA) and Tukey’s multiple comparison test were applied to evaluate significant differences at *p* < 0.05. All figures were generated with SciDAVis 2.7 (SciDAVis Development Team, open-source software).

### 2.9. Oxidative and Hydrolytic Properties Assessment of Flaxseed Oil

Acid value (AV) was determined according to the Chinese national standard GB 5009.229-2025 using the cold solvent indicator titration method (Method I) [40]. Briefly, approximately 2.5 g of oil sample was dissolved in 50 mL of a mixed solvent consisting of diethyl ether and isopropanol (1:1, *v*/*v*). After addition of 3–4 drops of phenolphthalein indicator, the solution was immediately titrated with 0.1 mol/L potassium hydroxide (KOH) solution until a pink color persisted for 15 s. A solvent blank was run simultaneously and used for correction. The acid value was calculated as the amount of KOH (in milligrams) required to neutralize the free fatty acids in 1 g of oil sample and expressed in mg KOH/g. The AV can be calculated using the following formula:

(4) $$AV\left(mg\;KOH/g\right)=\frac{(V-V_{0})\times c\times 56.1}{m}$$

where *V* and *V*_0_ are the volumes (mL) of KOH solution consumed in the titration of the sample and blank, respectively; *c* is the concentration of the standard KOH solution (mol/L); *m* is the sample mass (g); and 56.1 is the molar mass of KOH (g/mol). All measurements were performed in triplicate, and the results were reported as mean ± standard deviation.

Peroxide value (PV) was determined according to the Chinese national standard GB 5009.227-2023 using the indicator titration method (Method I) [41]. Briefly, approximately 2–3 g of oil sample was accurately weighed into an iodine flask and dissolved in 30 mL of a mixture of chloroform and glacial acetic acid (2:3, *v*/*v*). Then, 1 mL of saturated potassium iodide (KI) solution was added, and the flask was stoppered, shaken for 0.5 min, and allowed to stand in the dark for exactly 3 min. Subsequently, 100 mL of distilled water was added, and the liberated iodine was immediately titrated with standardized sodium thiosulfate solution (Na_2_S_2_O_3_). When the solution turned pale yellow, 1 mL of starch indicator solution (1 g/100 mL) was added, and titration was continued with vigorous shaking until the blue color just disappeared, which was taken as the endpoint. A solvent blank was run simultaneously, and the volume of thiosulfate consumed in the blank was checked to ensure it did not exceed 0.1 mL. The PV was calculated using the following equation:

(5) $$pV\left(g/100g\right)=\frac{(V-V_{0})\times c\times 0.1269}{m}\times 100$$

where *V* and *V*_0_ are the volumes (mL) of sodium thiosulfate solution consumed by the sample and blank, respectively; *c* is the concentration of the sodium thiosulfate standard solution (mol/L); *m* is the sample mass (g); and 0.1269 is the mass of iodine (in grams) equivalent to 1.00 mL of 1.000 mol/L sodium thiosulfate solution(g/mmol). The PV was expressed as g/100 g. All measurements were performed in triplicate, and the results were reported as mean ± standard deviation.

The contents of conjugated dienes (CD) and trienes (CT) in flaxseed oil were determined using a UV-Vis spectrophotometric method according to GB/T 22500-2024 [42]. Briefly, approximately 20 mg of oil sample was accurately weighed and dissolved in isooctane to a final volume of 10 mL. The absorbance of the resulting solution was measured at 232 nm and 268 nm using a UV-Vis spectrophotometer (UV-1900i, Shimadzu, Kyoto, Japan) with isooctane as the blank. The results were expressed as specific ex-tinction coefficients K_232_ and K_268_, calculated using the equation:

(6) $$K_{\lambda }=\frac{A_{\lambda }}{C\times L}$$

where *A_λ_* is the absorbance at wavelength *λ* (nm), *C* is the concentration of the oil solution (g/100 mL), and *L* is the cuvette path length (cm). All measurements were performed in triplicate, and the results were reported as mean ± standard deviation.

p-Anisidine value (p-AV) was determined according to the Chinese national standard GB/T 24304-2024 [43]. Briefly, approximately 1 g of oil sample was accurately weighed and diluted to 25 mL with isooctane in a volumetric flask. An aliquot of this solution was transferred to a glass-stoppered test tube, and a volume of p-anisidine reagent (2.5 g/L in glacial acetic acid) was added. The mixture was shaken and allowed to stand in the dark for exactly 10 min. The absorbance of the resulting solution was measured at 350 nm using a UV-Vis spectrophotometer against a solvent blank. The p-anisidine value was calculated using the following equation:

(7) $$p-AV=\frac{100QV}{m}\;[1.2\times (A_{1}-A_{2}-A_{0})]$$

where *V* is the volume of the test solution (mL, V = 25 mL), *m* is the sample mass (g); *Q* is the concentration of the sample in the test solution (g/mL, Q = 0.01 g/mL); *A*_0_ is the absorbance of the unreacted sample solution, *A*_1_ is the absorbance of the reacted sample solution, *A*_2_ is the absorbance of the reagent blank, and 1.2 is the correction factor for dilution of the test solution with 1 mL of p-anisidine reagent or glacial acetic acid solution. All measurements were performed in triplicate, and the results were reported as mean ± standard deviation.

The TOTOX (Total Oxidation Value) is essential indicators of oxidative status in edible oils and fats. These values are calculated using the PV and p-AV of edible fats and oils by the following formulas: TOTOX = 2PV + p-AV [44]. All measurements were performed in triplicate, and the results were reported as mean ± standard deviation.

Oxidative stability index (OSI) was determined using a Rancimat apparatus (892, Metrohm, Herisau, Switzerland) according to the AOCS Official Method Cd 12b-92 [45]. Briefly, approximately 2.50 g of oil sample was accurately weighed into the reaction vessel and subjected to an accelerated oxidation process at a constant temperature of 90 °C under a purified air flow of 10.0 L/h. The effluent air from the sample was bubbled through a vessel containing deionized water, and the conductivity of the water was continuously monitored. During oxidation, volatile organic acids (mainly formic and acetic acid) were released and dissolved in the deionized water, causing an increase in conductivity. The induction period was automatically recorded as the inflection point of the conductivity–time curve, and the OSI was expressed as the induction time (h). All measurements were performed in triplicate, and the results were reported as mean ± standard deviation.

## 3. Results

### 3.1. Lipid Compositions of the Experimental Lipids

**Figure 1:**
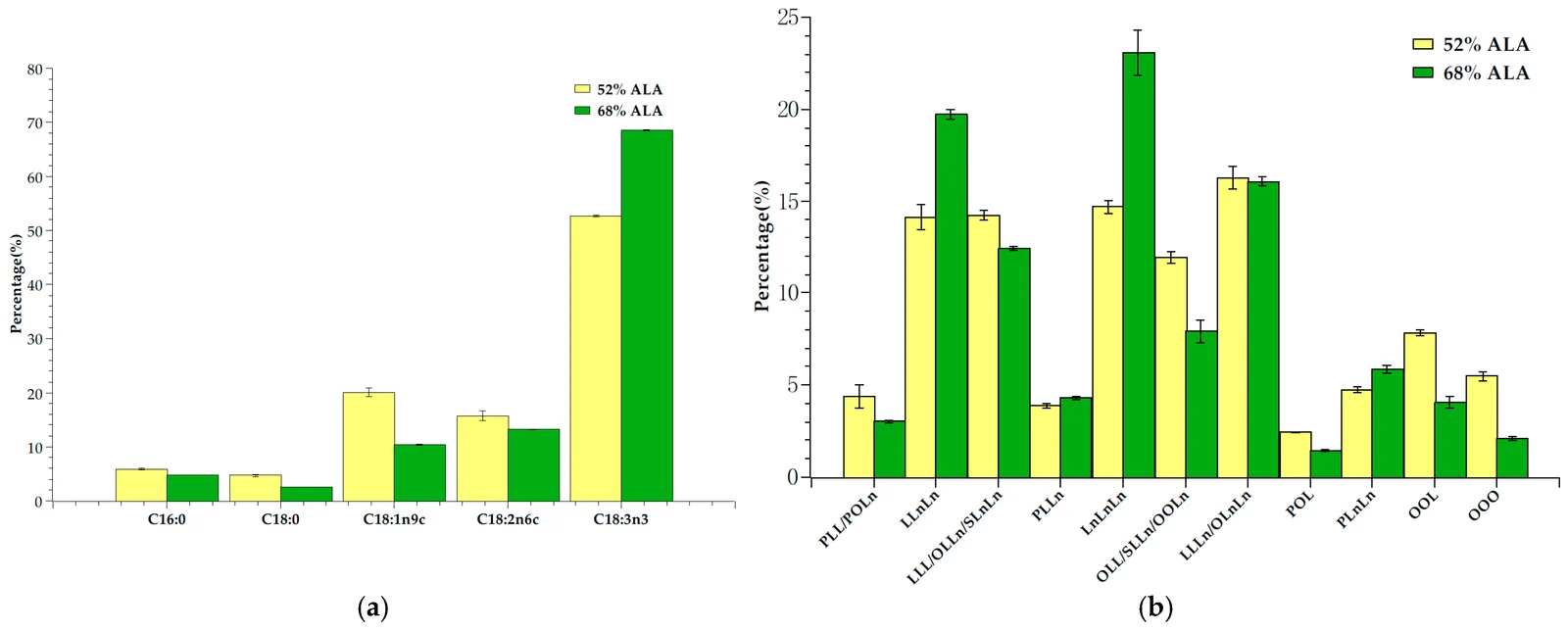
Lipid compositions of two flaxseed oils. (**a**) Fatty acid compositions; (**b**) TAG profiles. Data are presented as mean ± S.D., *n* = 3. (**a**) presents only the five dominant fatty acids for brevity, omitting minor components. Both oil samples contain seven additional minor fatty acids—including C14:0, C16:1, C20:0, and C20:1—which collectively account for approximately 0.9% in the 52% ALA oil and 0.6% in the 68% ALA oil.

The fatty acid and triacylglycerol profiles of two flaxseed oil samples are summarized in Figure 1, Tables S2 and S3; the compositions are expressed as percentages of total peak areas. As shown in Figure 1a, ALA is the predominant fatty acid in both oils, accounting for (52.8 ± 0.1%) and (68.5 ± 0.2%), respectively. Other major fatty acids include linoleic acid (14.5 ± 0.1%) and (13.2 ± 0.1%), oleic acid (19.5 ± 0.1%) and (10.3 ± 0.1%), stearic acid (4.4 ± 0.1%) and (2.6 ± 0.1%), and palmitic acid (5.5 ± 0.1%) and (4.8 ± 0.1%). The most notable difference between the two oils lies in their ALA content; therefore, they are designated as 52% ALA and 68% ALA throughout this study.

Figure 1b displays the triacylglycerol composition of the two oils. Compared to the 52% ALA oil, the 68% ALA oil exhibits significantly higher levels of LnLnLn and LLnLn, increasing from (14.7 ± 0.3%) to (23.1 ± 1.2%) and from (14.1 ± 0.7%) to (19.7 ± 0.3%), respectively. The fatty acid and triacylglycerol compositions of the two flaxseed oils differ significantly—not only in terms of ALA content but also in triacylglycerol structure. These compositional differences may influence digestive processes.

Prior to digestion experiments, the baseline hydrolytic and oxidative parameters of both cold-pressed, first-grade flaxseed oil samples were thoroughly characterized to ensure raw material freshness (Table S4). Both samples exhibited low acid values (AV ≤ 0.4 mg KOH/g), which were far below the Codex Alimentarius standard limit for cold-pressed edible oils (4.0 mg KOH/g). Furthermore, minimal total oxidation values (TOTOX < 5.2) were recorded, confirming their high initial quality and the absence of pre-existing rancidity. The slightly lower Oil Stability Index (OSI) of the 68% ALA sample (5.28 h vs. 5.97 h) reflects the inherently heightened susceptibility to oxidative degradation imparted by its greater degree of polyunsaturation.

### 3.2. Analysis of the In Vitro Digestibility and Digestion Process of Flaxseed Oil

Two analytical approaches were employed in this study to assess the *in vitro* digestibility and digestion process of flaxseed oil. NEFA release was analyzed using the pH-stat titration method, which monitors real-time lipolysis kinetics by titrating NaOH to neutralize protons released upon NEFA ionization. However, the detection efficacy of this method is inherently constrained by the ionization state of the fatty acids. Under acidic gastric conditions (pH 3.0–5.0), the generated fatty acids remain predominantly un-ionized (protonated); even under neutral intestinal conditions (pH 7.0), long-chain polyunsaturated fatty acids such as ALA fail to achieve complete ionization. This inherent limitation of pH-stat titration, which leads to a systematic underestimation of the overall lipolysis extent, has been well documented in standard protocols (Brodkorb et al., 2019; Chatzidaki et al., 2016) [29,46]. In contrast, triacylglycerol hydrolysis was evaluated using solvent extraction coupled with GC-FID, which quantifies the total NEFA (encompassing both ionized and un-ionized species) as well as the remaining lipids, including TAG, DAG, and MAG.

Therefore, the analysis of lipid lipolysis is structured into two perspectives: Section 3.2.1 focuses on real-time NEFA release kinetics during the intestinal phase following gastric digestion, whereas Section 3.2.2 assesses glyceride hydrolysis and total NEFA accumulation across the entire digestive process.

#### 3.2.1. NEFA Release

**Figure 2:**
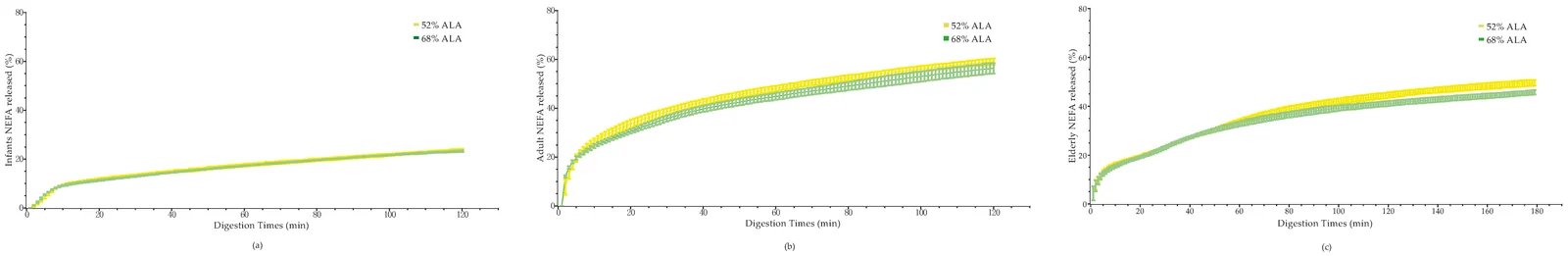
Levels of total NEFA released during the 120-min intestinal digestion phase of 52% and 68% ALA flaxseed oils across age-specific *in vitro* models: (**a**) infants model, (**b**) adult model, and (**c**) elderly model.

The pH-stat method was employed to systematically investigate the impact of intestinal digestion stages across different populations on the digestive behavior of two flaxseed oils with distinct ALA contents (52% and 68%). The resulting NEFA release profiles are presented in Figure 2 and Table 1.

The cumulative release of NEFA and the digestion kinetics exhibit a pronounced age-dependent pattern. The adult model demonstrates the highest digestive efficiency, with maximum NEFA release (59.1 ± 1.3%) and (56.4 ± 3.7%) and apparent rate constants (0.0310 s^−1^ and 0.0290 s^−1^) significantly exceeding those observed in infant and elderly models. Compared to adults, the elderly model shows weakened digestive function, characterized by markedly reduced NEFA release (49.6 ± 1.2%) and (45.7 ± 0.9%) and slower digestion rates (0.0227 s^−1^ and 0.0215 s^−1^). Although the infant model exhibits the lowest extent of NEFA release (23.7 ± 0.4%) and (23.3 ± 0.3%), its first-order kinetic rate constant (~0.026 s^−1^) is slightly higher than that of the elderly model. These differences highlight the regulatory influence of population-specific digestive enzyme activities and gastrointestinal microenvironments on lipid hydrolysis. Healthy adults possess fully developed and robust digestive systems, with optimal secretion of key enzymes such as pancreatic lipase and adequate bile salt release. Pancreatic lipase activity directly governs the rate of triacylglycerol hydrolysis, while bile salts facilitate the removal of hydrolysis products from lipid droplet surfaces, thereby exposing new substrate interfaces and enhancing enzymatic accessibility—synergistically enabling efficient lipid breakdown. In contrast, infants have immature digestive systems, and the elderly experience physiological decline, both resulting in substantially lower pancreatic lipase activity and bile salt concentrations compared to healthy adults. These deficiencies represent the primary physiological determinants underlying the reduced NEFA release in these populations.

In the same population-based *in vitro* digestion model, two flaxseed oils with differing ALA contents exhibited consistent digestive differences: the 52% ALA oil showed higher maximum NEFA release rates and rate constants than the 68% ALA oil across adult, infant, and elderly models. In the adult model, for instance, the maximum NEFA release rate of the 52% ALA oil (59.1%) was higher than that of the 68% ALA oil (56.4%), and its apparent rate constant (0.0310 s^−1^) exceeded that of the 68% ALA oil (0.0290 s^−1^). Although no statistically significant differences were observed, this trend was consistently maintained in both the elderly and infant models. The underlying mechanism may be attributed to the molecular structural properties of triacylglycerol. Previous studies have demonstrated that increased fatty acid unsaturation within triacylglycerol molecules is associated with reduced NEFA release [14,18]. ALA is an 18:3 n-3 long-chain polyunsaturated fatty acid (LCPUFA) that exhibits high conformational flexibility and steric hindrance when present at high concentrations. As the ALA content in triacylglycerol increases to 68%, greater molecular packing density may limit lipase accessibility, resulting in decreased NFFA release rates and lower cumulative release amounts.

#### 3.2.2. Triacylglycerol Hydrolysis Analysis

**Figure 3:**
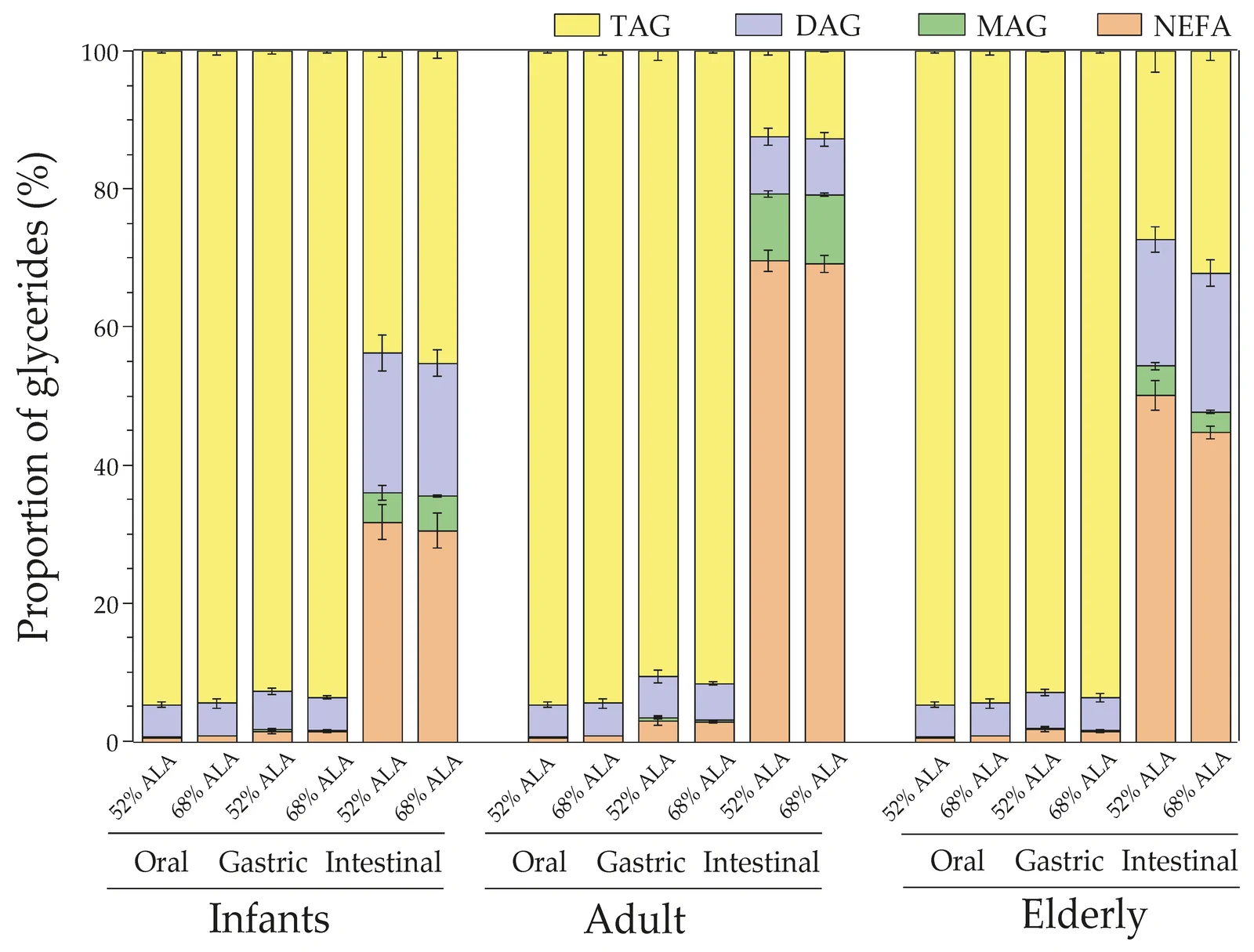
The relative contents of NEFA, MAG, DAG, and TAG in the final digestive products of different oils.

Figure 3 and Table S5 illustrates the compositional changes in TAG, DAG, monoacylglycerols (MAG), and NEFA across the oral, gastric, and intestinal phases of an *in vitro* digestion model applied to infant, adult, and elderly populations. These dynamics provide a clear visualization of how glyceride hydrolysis correlates with age-related digestive physiology.

In the oral phase, TAG levels remained above 95.0% across all groups regardless of ALA content, indicating minimal hydrolytic activity due to the absence of efficient lipolytic enzymes in this stage.

During the gastric phase, population-specific differences in lipolysis emerged, consistent with known variations in gastric lipase activity: in adults, TAG decreased to 90.6 ± 1.4% and NEFA increased to (3.0 ± 0.6%) for the 52% ALA group; infants exhibited less degradation, with TAG remaining at (92.8 ± 0.4%) and NEFA reaching only (1.4 ± 0.3%); similarly, elderly subjects showed limited hydrolysis, with TAG at (93.0 ± 0.2%) and NEFA at (1.7 ± 0.4%). Although the overall degree of lipolysis remains relatively low, this gradient change is still closely related to the physiological differences in the gastric fluid environment (such as lipase activity and pH value) among different populations, reflecting the characteristic change in digestive ability with age.

The intestinal phase represents the primary site of TAG hydrolysis. Here, marked intergroup differences were observed, driven by variations in digestive conditions: adults were exposed to high pancreatic lipase (2000 U/mL) and bile salt (10 mM) concentrations under optimal pH 7, resulting in a sharp decline in TAG to (12.4 ± 0.6%), a substantial increase in NEFA to (69.6 ± 1.5%), demonstrating efficient catalytic breakdown mediated by the pancreatic lipase–bile salt system. In contrast, infants experienced lower enzyme (90 U/mL) and bile salt (3 mM) levels at suboptimal pH 6.6, leading to incomplete hydrolysis, as evidenced by higher residual TAG (43.8 ± 0.9%) and lower NEFA release (31.7 ± 2.5%). Elderly subjects, exposed to intermediate enzyme and bile salt levels (1000 U/mL, 5 mM) over a prolonged period (3 h), displayed moderate TAG reduction (27.4 ± 3.0%) and NEFA generation (50.1 ± 2.1%), but notably elevated DAG levels (18.3 ± 1.9%), significantly exceeding those in adults (8.3 ± 1.2%), suggesting delayed clearance of hydrolysis intermediates due to reduced enzymatic efficiency.

When comparing ALA content effects within each population and phase, the 52% ALA group consistently yielded slightly higher maximum NEFA release than the 68% ALA group—for instance, in the elderly, 49.6% vs. 45.7%. This trend aligns with prior observations on NEFA release and supports the hypothesis that oils rich in ALA, with their higher degree of unsaturation, may be more resistant to complete lipolysis, likely due to structural or micellar solubilization challenges during digestion.

### 3.3. Analysis of the In Vitro Digestive Products of Flaxseed Oil

#### 3.3.1. ALA Bioaccessibility

*In vitro* digestion experiments revealed that the micellar bioaccessibility of ALA from flaxseed oil varies significantly across population groups and is modulated by lipid composition (Table 2).

Across the different population models, both the absolute amount of ALA in micelles and its bioaccessibility were consistently highest in the adult group. Taking the 52% ALA flaxseed oil as an example, the micellar ALA content was (64.83 ± 2.39 mg) in adults, significantly higher than that in the elderly (52.00 ± 1.18 mg) and infants (30.44 ± 2.55 mg). Similarly, ALA bioaccessibility reached (49.2 ± 1.8%) in adults, significantly exceeding that in the elderly (39.4 ± 0.9%) and infants (23.1 ± 1.9%). A parallel trend was observed with the 68% ALA flaxseed oil formulation. These findings indicate that the adult digestive environment enables the most efficient hydrolysis and micellar transfer of ALA. This trend is consistent with the previously described free fatty acid release kinetics, further underscoring the pivotal roles of digestive enzyme activity and bile salt secretion in lipid digestion.

Comparing the digestive performance of flaxseed oils containing 52% versus 68% ALA across infant, adult, and elderly populations, we observed that the higher-ALA oil (68%) increased the absolute amount of ALA in all three groups. However, bioaccessibility—defined as the fraction of total released ALA incorporated into mixed micelles—was consistently lower for the 68% ALA oil. Specifically, ALA release increased by 6.0%, 18.7%, and 18.7% in infants, adults, and the elderly, respectively, relative to the 52% ALA oil; yet corresponding bioaccessibility decreased by 3.6, 2.7, and 2.2 percentage points, respectively. These results indicate that although elevating the ALA content in flaxseed oil enhances total ALA liberation during digestion, the structural lability conferred by ALA’s three cis-double bonds renders it more susceptible to oxidative degradation in the gastrointestinal milieu and may impede efficient hydrolysis by pancreatic lipase.

#### 3.3.2. Changes in the Content of MDA

**Figure 4:**
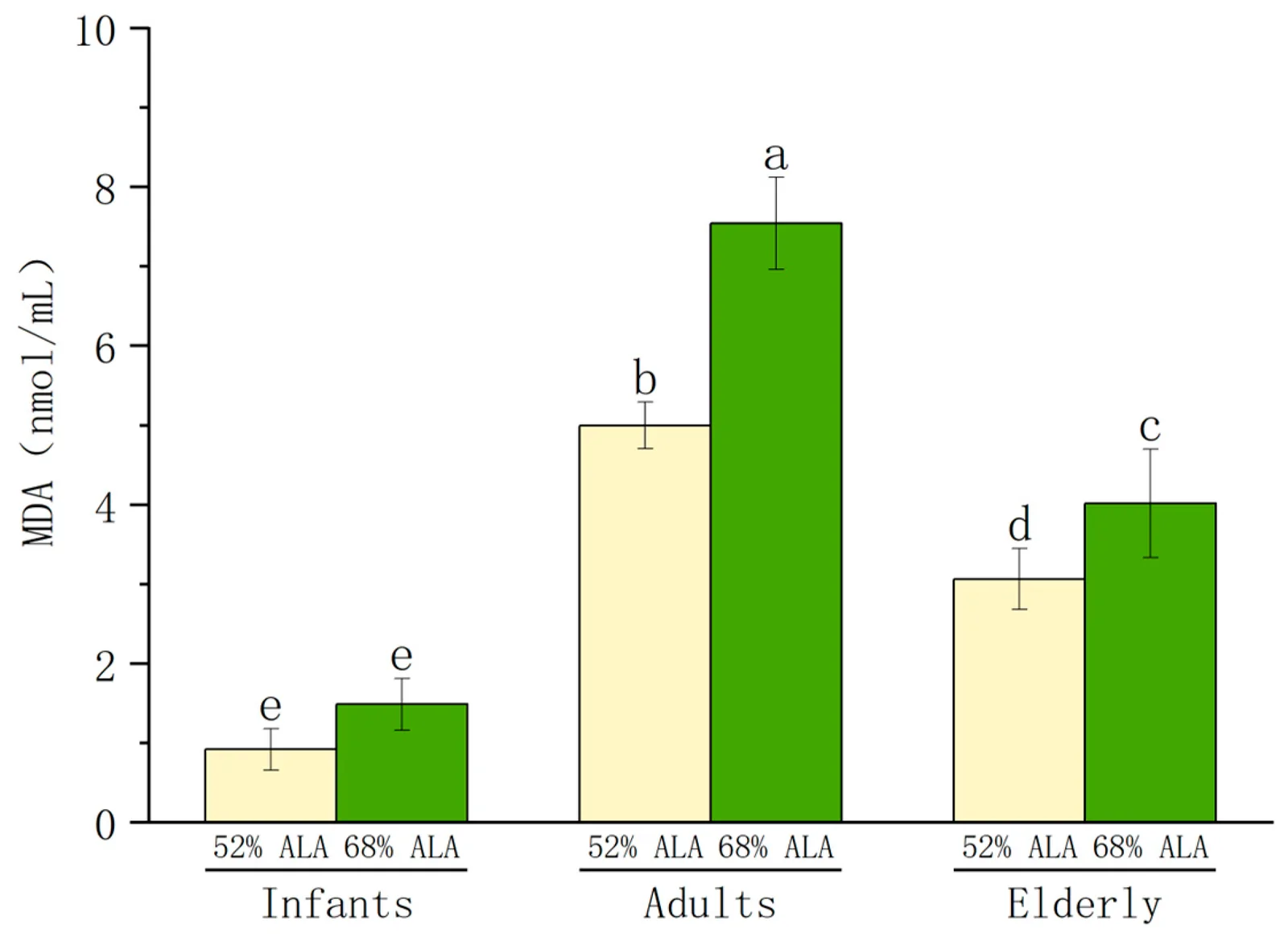
The characteristics of MDA formation following *in vitro* intestinal digestion of two flaxseed oils differing in ALA content. Different letters represent significant differences between different samples (*p* < 0.05).

MDA is a principal end-product of polyunsaturated fatty acid peroxidation and serves as one of the most widely used biomarkers of lipid peroxidation and oxidative stress [47]. As a highly reactive dicarbonyl compound, MDA can form covalent adducts with proteins and DNA, leading to protein dysfunction and mutagenic lesions [47,48]. Elevated MDA levels have been consistently observed in various pathological conditions, including cognitive impairment [49], cancer [48], neurodegenerative diseases [50], and atherosclerosis [51], underscoring its pathophysiological relevance. Given its chemical reactivity, broad disease association, and well-established detection methods, MDA was selected as the primary biomarker for assessing oxidative stress in the present study. Figure 4 illustrates the accumulation features of MDA across different population-based digestion models and varying ALA contents in flaxseed oil.

Significant age-dependent differences in MDA generation were observed among the digestion models. The highest MDA levels were detected in the adult group (5.00 ± 0.29 nmol/mL and 7.54 ± 0.58 nmol/mL), indicating the most pronounced lipid peroxidation under simulated adult digestive conditions. This finding correlates directly with the superior digestive efficiency and rapid NEFA release rate in adults: enhanced lipolysis increases the exposure of unsaturated fatty acids to pro-oxidative elements such as the aqueous phase, digestive enzymes, and metal ions, thereby accelerating oxidative reactions. In contrast, the infant group exhibited the lowest MDA concentration (0.92 ± 0.26 nmol/mL and 1.49 ± 0.32 nmol/mL), likely due to lower enzymatic activity and slower NEFA liberation, which limit interactions between lipids and oxidative triggers. The elderly group displayed intermediate MDA levels (3.06 ± 0.38 nmol/mL and 4.02 ± 0.68 nmol/mL), suggesting a moderate oxidative stress environment during digestion. These results collectively demonstrate that while enhanced lipid digestion efficiency promotes the release of nutrients, it simultaneously increases the exposure of free unsaturated fatty acids to pro-oxidative conditions in the digestive milieu, thereby exacerbating their oxidation risk.

When comparing two flaxseed oils differing in ALA content, the 68% ALA formulation consistently yielded higher MDA levels than the 52% ALA counterpart across all models, with a statistically significant difference observed in the adult and elderly models (*p* < 0.05). This difference is primarily attributed to the molecular structural features of ALA—as an ω-3 polyunsaturated fatty acid with three carbon–carbon double bonds, its high degree of unsaturation renders it particularly susceptible to oxidative degradation. Under identical digestive conditions, flaxseed oil containing 68% ALA releases a greater amount of free ALA (Section 3.3.1), and increased exposure of free ALA significantly enhances its likelihood of interaction with pro-oxidative factors in the digestive milieu, ultimately increasing the formation of secondary oxidation products such as MDA. Therefore, when evaluating the digestive properties of ALA-rich flaxseed oil, oxidative stability should be considered a critical parameter for comprehensively assessing the nutritional quality of digested lipids, as higher ALA values do not necessarily indicate better performance.

## 4. Discussion

This study revealed significant findings across different *in vitro* digestion models representing diverse human populations. Within the same population, increasing the ALA content in flaxseed oil from 52% to 68% led to a higher absolute release amount of ALA (NEFA-ALA + MAG-ALA) yet was accompanied by a slight reduction in ALA release rate and a concurrent increase in MDA production. This pattern of interrelated outcomes provides critical insights into the digestive behavior and oxidative stability of high-polyunsaturated fatty acid (PUFA) oils under simulated gastrointestinal conditions.

From a nutritional standpoint, despite a slight reduction in release rate, the 68% ALA group exhibited a higher absolute release of ALA compared to the 52% ALA group, attributable to the greater initial concentration of ALA in the substrate. Considering the prevalent insufficiency of omega-3 fatty acid intake [52,53], a higher ALA supply is theoretically better positioned to meet the body’s requirements for polyunsaturated fatty acids. However, the simultaneous increase in MDA levels indicates that the high ALA content also brings a greater risk of oxidation. This not only may reduce the final availability of active ALA, but its oxidative products may also have potential negative impacts on intestinal health. Under gastrointestinal conditions, the accumulation of free MDA could potentially pose a profound threat, as it is known to propagate through chain reactions and form covalent adducts with luminal macromolecules, proteins, and nucleic acids, thereby having the potential to disrupt the integrity of the healthy gut microenvironment and exacerbate localized oxidative stress [54,55,56,57,58]. Although the MDA accumulation in this study was monitored within an *in vitro* digestion model, and actual human health outcomes depend on the complex interplay between matrix protection mechanisms and the underlying mucosal defense systems, these findings nonetheless indicate that the heightened chemical susceptibility of ALA to lipid peroxidation warrants cautious risk management [59]. Moreover, polyunsaturated fatty acids (PUFAs) are also susceptible to oxidation following absorption, particularly during absorptive processing within small intestinal epithelial cells and subsequent systemic circulation, which may further compromise the overall bioavailability of intact ω-3 fatty acids [60]. Certainly, while ALA is inherently prone to MDA generation as a polyunsaturated fatty acid, this vulnerability does not completely negate the practical utility and fundamental dietary necessity of flaxseed oil as a primary plant-based source of essential omega-3 fatty acids. This dual impact underscores that lipid peroxidation is a critical limiting factor, determining whether a high-ALA oil can successfully function as a safe nutritional vehicle or is merely restricted to industrial applications such as paints and varnishes. Therefore, the key to enhancing the real-world value of highly unsaturated vegetable oils lies in tailoring strategies to population-specific physiological characteristics, carefully balancing high ALA release with highly efficient antioxidant protection. This proposed equilibrium between nutritional supply and oxidative defense is more critically dependent on the unique digestive physiological characteristics of distinct populations. For adults, the mature digestive system—characterized by high pancreatic lipase activity and sufficient bile salt concentration—enables efficient hydrolysis of glycerides. Consequently, the higher absolute release of ALA observed in the 68% ALA group translates into tangible nutritional benefits. However, the concurrent rise in MDA levels indicates an increased oxidative burden during digestion. Long-term consumption of such high-ALA oils may lead to excessive accumulation of lipid oxidation products in the gastrointestinal tract, potentially disrupting the intestinal microenvironment or elevating systemic oxidative stress. Therefore, high-ALA flaxseed oil suitable for adult consumption should be enriched with antioxidant compounds such as vitamin E or polyphenols to mitigate lipid peroxidation during digestion [61,62]. For infants, although both the ALA release rate and MDA levels are the lowest, suggesting a protective effect conferred by their milder digestive conditions, the extremely low ALA release also poses a risk of inadequate nutrient absorption. For elderly groups, age-related declines in digestive function may limit their capacity to process high-ALA oils effectively, while the elevated MDA levels further exacerbate digestive and oxidative strain. Therefore, for infants and the elderly with insufficient digestive function, greater emphasis should be placed on enhancing the bioavailability of ALA and mitigating lipid oxidation risk, rather than solely pursuing high ALA content. Strategies such as the use of structured lipids or optimized emulsification systems may effectively support these objectives [21,22,63]. It is worth noting that the population-specific ALA intake strategies from flaxseed oil are fundamentally premised on a normal gastrointestinal physiological baseline. In clinical scenarios involving individuals with gastrointestinal dysfunction—such as severe achlorhydria, exocrine pancreatic insufficiency, or impaired mucosal integrity—the standard kinetics of lipolysis and structural micellization are substantially disrupted [64,65]. Consequently, generalized suggestions may prove inadequate for these populations, necessitating highly personalized nutritional interventions. Most studies have primarily focused on the nutritional benefits of high ALA content in flaxseed oil, whereas this study highlights the “oxidative cost associated with high ALA during digestion” and the “modulating role of population-specific digestive physiology.” When assessing the ALA level in flaxseed oil, a balance should be struck between nutritional supply and oxidation risk, and it should also match the digestive physiological characteristics of the target population. It is necessary to clarify that while standardized static *in vitro* digestion models provide a reliable platform to determine the intrinsic lipid lipolysis mechanics and concomitant chemical peroxidation susceptibility under specific enzymatic and chemical microenvironments, their linear extrapolation to living organisms possesses distinct boundaries, particularly when expanding the analysis to encompass the highly complex and systemic physiological profiles of diverse age cohorts. Compared to alternative systems such as everted intestinal sacs—which are traditionally used to evaluate downstream epithelial permeability and active mucosal transport—the *in vitro* digestion system employed here is well-suited for assessing the initial stages of lipid fate and chemical stability. However, this static setup inherently lacks the crucial clearance mechanisms provided by mucosal absorption, which may lead to an overestimation of metabolite accumulation, such as MDA, relative to actual *in vivo* scenarios [66,67]. Future research should integrate Caco-2 cell models or animal experiments to further validate the “nutrient–oxidation” trade-off of ALA from flaxseed oil under physiological conditions. Additionally, investigating the inhibitory effects of various antioxidants—such as plant polyphenols—on MDA accumulation during the digestion of high-ALA flaxseed oil could provide more direct evidence for optimizing the formulation of functional lipids.

## 5. Conclusions

This study employed an *in vitro* simulation of the digestive environments of infants, adults and the elderly to systematically compare the digestive characteristics of two flaxseed oils differing in ALA content. First, digestive efficiency exhibited marked population-dependent variation. Adults showed the highest digestive performance, as evidenced by greater NEFA release, glyceride hydrolysis, and ALA bioaccessibility, whereas infants exhibited the lowest efficiency, with the elderly displaying intermediate levels. These differences are closely associated with variations in digestive enzyme activity, bile salt secretion and gastrointestinal conditions across age groups [29,30]. Second, higher ALA content in flaxseed oil increases the absolute release of ALA but also elevates oxidative risk. Within each population, the 68% ALA flaxseed oil exhibited lower lipolysis rates and NEFA release compared to the 52% ALA oil; although the former achieved higher absolute ALA release, it also generated significantly more malondialdehyde (MDA), particularly in the adult model. This indicated that oils rich in highly unsaturated fatty acids face pronounced oxidative challenges under physiological digestive conditions. These findings highlight that the development of ALA-enriched oils for specific populations requires a balanced consideration of digestive efficiency and oxidative stability to ensure both nutritional efficacy and safety. The results provided experimental support for tailored lipid nutrition design and personalized dietary strategies, and offer insights into the application of highly unsaturated plant oils in age-specific or physiologically vulnerable populations.

## Figures and Tables

**Table 1:** The maximum release amount of fatty acids and the release kinetics equation. * Data are presented as mean ± S.D., *n* = 3.

Population	Sample	NEFA Maximum Released %	Kinetics Equation	Apparent Rate Constant (s^−1^)	Linear R_adj_^2^
Infants	52% ALA	23.7 ± 0.4	y = −0.0269x + 0.0306	0.0269	0.881
	68% ALA	23.3 ± 0.3	y = −0.0261x − 0.0007	0.0261	0.901
Adults	52% ALA	59.1 ± 1.3	y = −0.0310x − 0.0690	0.0310	0.902
	68% ALA	56.4 ± 3.7	y = −0.029x − 0.0767	0.0290	0.898
Elderly	52% ALA	49.6 ± 1.2	y = −0.0227x + 0.0147	0.0227	0.922
	68% ALA	45.7 ± 0.9	y = −0.0215x + 0.0152	0.0215	0.936

**Table 2:** The absolute release of ALA in micelles and ALA bioaccessibility. * Data are presented as mean ± S.D., *n* = 3.

Population	Sample	ALA Absolute Release Amount (mg)	ALA Bioaccessibility (%)
Infants	52% ALA	30.44 ± 2.55	23.1 ± 1.9
	68% ALA	32.26 ± 2.39	19.5 ± 1.4
Adults	52% ALA	64.83 ± 2.39	49.2 ± 1.8
	68% ALA	76.96 ± 2.31	46.5 ± 1.4
Elderly	52% ALA	52.00 ± 1.19	39.4 ± 0.9
	68% ALA	61.72 ± 1.99	37.3 ± 1.2

## Data Availability

The original contributions presented in this study are included in the article/Supplementary Materials. Further inquiries can be directed to the corresponding author.

## Supplementary Materials

The following supporting information can be downloaded at: https://www.mdpi.com/article/10.3390/foods15162891/s1. Table S1. Triacylglycerol composition of flaxseed oil analyzed by UPLC–HRMS. Table S2. Fatty acid composition of the two flaxseed oils. Table S3. Triacylglycerol composition of the two flaxseed oils. Table S4. Oxidative stability and quality characteristics of two flaxseed oils. Table S5. Relative contents of NEFA, MAG, DAG, and TAG (%) in the final digestive products of two flaxseed oils.
