# Supplementary material for: Is Higher α-Linolenic Acid Content Preferable? Assessing Digestive Fate of Flaxseed Oil in In Vitro Digestion Models of Infants, Adults and the Elderly
Source: Foods. 2026 Aug 18;15(16):2891. doi: 10.3390/foods15162891 (PMC13512170; doi:10.3390/foods15162891)
Supplement: Supplementary file 1 [file foods-15-02891-s001.zip › foods-4451155-supplementary-english.pdf]

**Table S1.** Triacylglycerol composition of flaxseed oil analyzed by UPLC–HRMS

| Triacylglycerol* | Fatty acyl chain | Formula | m/z [M+NH4]+ |
|---|---|---|---|
| **PLnLn** | 16:0/18:3/18:3 | $C_{55}H_{94}O_6$ | 868.7389 |
| **PLLn** | 16:0/18:2/18:3 | $C_{55}H_{96}O_6$ | 870.7545 |
| **PLL/POLn** | 16:0/18:2/18:2; 16:0/18:1/18:3 | $C_{55}H_{98}O_6$ | 872.7702 |
| **POL** | 16:0/18:1/18:2 | $C_{55}H_{100}O_6$ | 874.7858 |
| **LnLnLn** | 18:3/18:3/18:3 | $C_{57}H_{92}O_6$ | 890.7232 |
| **LLnLn** | 18:2/18:3/18:3 | $C_{57}H_{94}O_6$ | 892.7389 |
| **LLLn/OLnLn** | 18:2/18:2/18:3; 18:1/18:3/18:3 | $C_{57}H_{96}O_6$ | 894.7545 |
| **LLL/OLLn/SLnLn** | 18:2/18:2/18:2; 18:1/18:2/18:3; 18:0/18:3/18:3 | $C_{57}H_{98}O_6$ | 896.7702 |
| **OLL/SLLn/OOLn** | 18:1/18:2/18:2; 18:0/18:2/18:3; 18:1/18:1/18:3 | $C_{57}H_{100}O_6$ | 898.7858 |
| **OOL** | 18:1/18:1/18:2 | $C_{57}H_{102}O_6$ | 900.8015 |
| **OOO** | 18:1/18:1/18:1 | $C_{57}H_{104}O_6$ | 902.8171 |

*P, Palmitic acid; S- Stearic acid; O, Oleic acid; L, Linoleic acid; Ln, Linolenic acid. Semicolons (;) separate co-eluting isomeric species that could not be resolved by UPLC-HRMS. [M+NH4]+ refers to the ammonium adduct ion.

**Table S2**. Fatty acid composition of the two flaxseed oils

| Fatty acid | 52% ALA % | 68% ALA % |
|---|---|---|
| **Main fatty acids** | | |
| **C16:0** | 5.9 ± 0.1 | 4.8 ± 0.1 |
| **C18:0** | 4.7 ± 0.1 | 2.6 ± 0.1 |
| **C18:1n9c** | 20.0 ± 0.1 | 10.3 ± 0.1 |
| **C18:2n6c** | 15.7 ± 0.2 | 13.2 ± 0.2 |

| | | |
|---|---|---|
| **C18:3n3** | 52.7 ± 0.2 | 68.5 ± 0.2 |
| **Total amount of main fatty acids** | 99.1 | 99.4 |
| **Other fatty acids** | | |
| **C14:0** | 0.050 ± 0.001 | 0.059 ± 0.001 |
| **C16:1** | 0.077 ± 0.001 | 0.059 ± 0.001 |
| **C20:0** | 0.196 ± 0.001 | 0.085 ± 0.001 |
| **C20:1** | 0.253 ± 0.001 | 0.123 ± 0.001 |
| **C22:0** | 0.140 ± 0.001 | 0.106 ± 0.001 |
| **C23:0** | 0.058 ± 0.001 | 0.042 ± 0.001 |
| **C24:0** | 0.131 ± 0.001 | 0.106 ± 0.001 |
| **Total of other fatty acids** | 0.905 | 0.580 |
| **Total** | 100.0 | 100.0 |

**Table S3**. Triacylglycerol composition of the two flaxseed oils

| **Triacylglycerol*** | **52%ALA %** | **68%ALA %** |
|---|---|---|
| **PLL/POLn** | 4.4 ± 0.6 | 3.0 ± 0.1 |
| **LLnLn** | 14.1 ± 0.7 | 19.7 ± 0.3 |
| **LLL/OLLn/SLnLn** | 14.2 ± 0.3 | 12.4 ± 0.1 |
| **PLLn** | 3.9 ± 0.1 | 4.3 ± 0.1 |
| **LnLnLn** | 14.7 ± 0.3 | 23.1 ± 1.2 |
| **OLL/SLLn/OOL** | 12.0 ± 0.3 | 7.9 ± 0.6 |
| **LLLn/OLnLn** | 16.3 ± 0.6 | 16.1 ± 0.2 |
| **POL** | 2.4 ± 0.1 | 1.4 ± 0.1 |
| **PLnLn** | 4.8 ± 0.2 | 5.9 ± 0.2 |
| **OOL** | 7.8 ± 0.2 | 4.1 ± 0.3 |
| **OOO** | 5.5 ± 0.2 | 2.1 ± 0.1 |

| **Total** | 100.0 | 100.0 |
|---|---|---|

*P, Palmitic acid; S- Stearic acid; O, Oleic acid; L, Linoleic acid; Ln, Linolenic acid.

**Table S4.** Oxidative stability and quality characteristics of two flaxseed oils

| Sample | AV（mg KOH/g） | PV（g/100g） | p-AV | TOTOX | CD ($K_{232}$) | CT ($K_{268}$) | OSI (h) |
|---|---|---|---|---|---|---|---|
| **52% ALA** | 0.20 ± 0.01 | 0.06 ± 0.02 | 4.57 ± 0.31 | 4.97 ± 0.32 | 5.31 ± 0.14 | 0.29 ± 0.01 | 5.97 ± 0.13 |
| **68% ALA** | 0.40 ± 0.01 | 0.06 ± 0.01 | 4.36 ± 0.56 | 5.16 ± 0.56 | 4.37 ± 0.04 | 0.42 ± 0.02 | 5.28 ± 0.14 |

**Table S5.** Relative contents of NEFA, MAG, DAG, and TAG (%) in the final digestive products of two flaxseed oils

| Age group | Sample | Digestive phase | NEFA % | MAG % | DAG % | TAG % |
|---|---|---|---|---|---|---|
| **Infant** | **52% ALA** | Oral | 0.5 ± 0.1 | 0.1 ± 0.04* | 4.3 ± 1.0 | 95.1 ± 1.3 |
| | | Gastric | 1.4 ± 0.2 | 0.19 ± 0.09* | 5.6 ± 1.2 | 92.8 ± 1.3 |
| | | Intestinal | 31.7 ± 2.5 | 4.2 ± 1.1 | 20.2 ± 2.6 | 43.8 ± 0.9 |
| | **68% ALA** | Oral | 0.6 ± 0.1 | 0.1 ± 0.03* | 4.3 ± 0.8 | 95.1 ± 1.0 |
| | | Gastric | 1.4 ± 0.2 | 0.20 ± 0.05* | 4.8 ± 0.9 | 93.6 ± 1.0 |
| | | Intestinal | 30.5 ± 2.5 | 5.0 ± 0.2 | 19.2 ± 1.9 | 45.3 ± 1.0 |
| **Adult** | **52% ALA** | Oral | 0.5 ± 0.1 | 0.1 ± 0.02* | 4.3 ± 0.9 | 94.2 ± 1.0 |
| | | Gastric | 3.0 ± 0.6 | 0.5 ± 0.2 | 5.9 ± 0.9 | 90.6 ± 1.4 |
| | | Intestinal | 69.6 ± 1.6 | 9.7 ± 0.4 | 8.3 ± 1.2 | 12.4 ± 0.7 |
| | **68% ALA** | Oral | 0.6 ± 0.2 | 0.1 ± 0.03* | 4.3 ± 1.0 | 95.0 ± 1.1 |
| | | Gastric | 2.8 ± 0.5 | 0.3 ± 0.02* | 5.3 ± 0.7 | 91.6 ± 0.3 |
| | | Intestinal | 69.1 ± 1.3 | 10.0 ± 0.2 | 8.1 ± 1.0 | 12.8 ± 0.2 |

| | | | | | | |
|---|---|---|---|---|---|---|
| **Elderly** | **52% ALA** | Oral | 0.6 ± 0.1 | 0.1 ± 0.02* | 4.2 ± 0.9 | 95.1 ± 1.2 |
| | | Gastric | 1.7 ± 0.4 | 0.2 ± 0.03* | 5.1 ± 0.5 | 93.0 ± 0.2 |
| | | Intestinal | 50.1 ± 2.1 | 4.3 ± 0.5 | 18.3 ± 1.9 | 27.4 ± 3.0 |
| | **68% ALA** | Oral | 0.6 ± 0.1 | 0.1 ± 0.02* | 4.2 ± 1.0 | 95.1 ± 1.2 |
| | | Gastric | 1.4 ± 0.2 | 0.2 ± 0.04* | 4.7 ± 0.6 | 93.8 ± 0.4 |
| | | Intestinal | 44.7 ± 0.9 | 3.0 ± 0.2 | 20.1 ± 1.9 | 32.2 ± 1.3 |

* Values are presented with two decimal places due to the small magnitude of the standard deviation (SD)
